# Validation of the Health Literacy in Dentistry Scale (HeLD-14) in the Greek Language

**DOI:** 10.7759/cureus.97451

**Published:** 2025-11-21

**Authors:** Maria Bakali, Maria Saridi, Constantinos Togas, Ourania S Kotsiou, Evangelos C Fradelos, Aristomenis I Syngelakis, Dimitrios Papagiannis

**Affiliations:** 1 Laboratory of Public Health and Adults Immunization, Department of Nursing, University of Thessaly, Larissa, GRC; 2 Department of Nursing, University of Thessaly, Larissa, GRC; 3 Department of Psychology, Panteion University, Athens, GRC; 4 Laboratory of Human Pathophysiology, Department of Nursing, University of Thessaly, Larissa, GRC; 5 Faculty of Dentistry, National and Kapodistrian University, Athens, GRC; 6 School of Dentistry, European University Cyprus, Nicosia, CYP; 7 Department of Neurology, Medical School, University of Cyprus, Nicosia, CYP

**Keywords:** greek, health literacy, held-14, oral health, psychometrics, validation

## Abstract

Objectives: To translate and culturally adapt the Health Literacy in Dentistry Scale (HeLD-14) into the Greek language.

Methods: Following the official translation of the questionnaire, a cross-sectional study was carried out involving 333 participants from the general Greek population. The self-administered questionnaire, completed both in paper-and-pencil format and electronically, included sections on demographic information and oral health-related data, along with the translated versions of the Health Literacy in Dentistry Scale (HeLD-14) and the European Health Literacy Survey Questionnaire (HLS-EU-Q16). The study was conducted from March to July 2025, and data were analyzed using Statistical Product and Service Solutions (SPSS, version 29; IBM SPSS Statistics for Windows, Armonk, NY).

Results: Exploratory factor analysis indicated that the HeLD-14 operates as a single-factor instrument, with all items showing factor loadings above 0.40 on this factor. The 14-item scale demonstrated strong internal consistency, with Cronbach’s alpha of 0.879 and McDonald’s ω of 0.877. Additionally, there was a positive and statistically significant correlation (r = 0.424, p < 0.001) between HeLD-14 scores and the total scores of the HLS-EU-Q16, supporting the concurrent validity of the Greek version of the HeLD-14.

Conclusions: The HeLD-14 is a reliable and valid tool for evaluating various aspects of oral health literacy among Greek-speaking adults. Its concise format and ease of use, along with strong psychometric properties, make it suitable for evaluating oral health literacy in both public health research and routine clinical practice.

## Introduction

Health literacy is the capacity of a person to obtain, comprehend, and interpret essential health information and services required to make informed health choices [1]. It includes skills such as reading and comprehending written materials, effectively communicating health information, understanding how to use the healthcare system, and achieving the best possible health results [2].

Oral health literacy is a key subset of health literacy, introduced in the Healthy People 2010 initiative [3]. It is considered a fundamental factor influencing oral health and involves the ability to access, understand, and use essential oral health information. It also includes the capacity to navigate oral health services and make informed decisions related to oral healthcare [4].

Oral diseases affect not only the mouth but also overall body health. Dental and oral conditions can trigger or worsen systemic illnesses, such as endocarditis, arthritis, diabetes, and nephritis [5]. Consequently, oral health literacy is essential for improving oral health.

Patients with low oral health literacy have poorer oral health knowledge, worse oral health status, and lower quality of life [4,6]. They often miss appointments, avoid preventive care, and do not follow dental advice, leading to worse oral health outcomes and higher treatment costs [7].

Only a few tools exist to measure oral health literacy, allowing for more accurate data collection. Key instruments include the Rapid Estimate of Adult Literacy in Dentistry (REALD-30 items) [8], the Rapid Estimate of Adult Literacy in Dentistry (REALD-99 items) [9], the Hong Kong Rapid Estimate of Adult Literacy in Dentistry [10], the Rapid Estimate of Adult Literacy in Medicine and Dentistry (REALM-D) [11], the Test of Functional Health Literacy in Dentistry (TOFHLiD) [12], the Oral Health Literacy Instrument (OHLI) [13], and the Comprehensive Measure of Oral Health Knowledge (CMOHK) [14]. Newer tools include the Oral Health Literacy-Adult Questionnaire (OHL-AQ) [15] and the Health Literacy in Dentistry Scale (HeLD-29) with its shorter version, HeLD-14 [16].

The HeLD-29, adapted from the Health Literacy Measurement Scale (HeLMS), measures seven dimensions of oral health literacy: receptivity, comprehension, support, economic barriers, access, communication, and utilization [17]. It was first applied among Indigenous Australians, showing good validity and reliability, and later tested in a pilot study with Indonesian undergraduate students [4,18].

The HeLD-14 is a shortened version of the HeLD-29, designed to make oral health literacy assessment easier. It maintains strong validity and reliability while reducing respondent burden, making it more practical for both clinical and research use [16,19].

The HeLD-14 has been validated in various populations worldwide, including samples from the United States [20], China [21], Thailand [22], Brazil [19], Peru [23], Arab countries [24], and Colombia [25]. Most studies confirm its strong internal consistency and, in many cases, satisfactory validity.

Studies on the factor structure of the HeLD have produced mixed results, with dimensions ranging from one to seven [20]. For the HeLD-29, Jones et al. found a unidimensional solution among Indigenous Australians but provided limited analysis [4]. Their principal components analysis showed one dominant factor (explaining 37.4% of variance) plus six smaller factors, supported by scree plot evidence, indicating a possible multidimensional structure. Low yet significant correlations among the seven variables added to the uncertainty about the true dimensionality of oral health literacy [4].

Factor analysis of the HeLD-14 revealed six components, as the original "utilization" domain was merged with "communication" [16].

Findings on the HeLD-14’s factor structure vary across studies. The Peruvian version of the HeLD-14 showed a multifactorial structure [23]. Similarly, two Brazilian validation studies comparing the HeLD-29 and HeLD-14 also found a multifactorial structure: one with 535 older adults from primary care centers and a dental clinic [19], and another with 603 adults recruited through the same methods [26].

US and Chinese validations reported a unidimensional model [20,21], while Thai and Brazilian studies often identified six factors, though some inconsistencies in factor loadings were noted [22,27,28].

To date, no validated instruments assessing this construct have been available for Greek-speaking populations. A Greek version of the HeLD-14 is necessary, as there is a scarcity of studies and insufficient data on oral health literacy in Greece, along with a lack of organized initiatives promoting it. This tool can effectively measure the level of oral health literacy among the Greek population and support researchers and health professionals in developing targeted educational and awareness programs in this field. Therefore, this study aimed to translate the HeLD-14 into Greek and evaluate its factor structure, internal consistency, and concurrent validity among Greek adults.

## Materials and methods

Translation procedure of the HeLD-14

The HeLD-14 was translated into Greek following standard protocols [29]: two forward translations, review and harmonization by a bilingual expert, and backward translation by two bilingual experts to ensure accuracy. The Greek version was pilot-tested with 15 adults to assess clarity, leading to final revisions. Almost all questions were easy to understand. Only minor difficulty was encountered with Item 6 (“Are you able to ask for support for a dental appointment?”), for which particular attention was given to achieving the most accurate and culturally appropriate translation into Greek. Average completion time was about two minutes.

Participants

The study included Greek-speaking adults who provided informed consent, excluding anyone unable to complete the questionnaire. The final sample consisted of 333 participants aged 18-85 years (mean age: 44.02 ± 13.03, minimum = 18, maximum = 85). Given that an item-to-participant ratio of 1:5 to 1:10 is considered ideal for factor analysis, and the original HeLD-14 contains 14 items, the study’s sample size of 33 participants was deemed adequate for factor analysis. Further demographic and clinical information can be found in Table 1.

**Table 1:** Demographic and medical characteristics of the study sample

Demographic/medical characteristics	Frequency	%
Gender		
Male	96	28.83
Female	235	70.57
Other	2	0.60
Marital/relationship status		
Single	63	18.92
Married	221	66.37
In a romantic relationship	24	7.21
Separated	6	1.80
Divorced	12	3.60
Widow/widower	7	2.10
Educational level		
Primary school	6	1.80
Secondary school	11	3.30
High school	81	24.32
University/technological educational institute student	19	5.71
Graduate of a university or a technological educational institute	121	36.34
Master of Science degree holder	84	25.23
Doctorate degree holder	11	3.30
Job		
Housewife	26	7.81
Unemployed	23	6.91
Manual labor worker	3	0.90
Student	15	4.51
Farmer	4	1.20
Civil servant	89	26.73
Private employee	91	27.33
Freelancer	51	15.32
Pensioner	31	9.
Place of residence		
City	224	67.27
Town	86	25.83
Village	23	6.
Self-assessment of oral health		
Very poor	2	0.60
Poor	9	2.71
Average	82	24.70
Good	187	56.33
Very good	52	15.66
Visit to the dentist		
Regularly (e.g. every six months)	158	47.45
Only when there is a problem	174	52.25

Measures

Demographic and Oral Health-Related Information

Participants reported on gender, age, marital status, education, occupation, residence, self-assessed oral health, and frequency of dental visits (regular or problem-driven).

Health Literacy in Dentistry Scale (HeLD-14)

It measures oral health literacy across seven domains - receptivity, understanding, support, economic barriers, access, communication, and utilization - with two items per domain [4]. Each item asks about the difficulty in performing dental-related tasks and is scored from 0 (“unable to do”) to 4 (“without difficulty”), giving a total score range of 0-56. Higher scores indicate better oral health literacy [16].

European Health Literacy Survey Questionnaire (HLS-EU-Q16)

It is a 16-item self-administered questionnaire assessing general health literacy, focusing on accessing, understanding, and evaluating health information [30-32]. Items are rated on a four-point scale ranging from "very difficult" to "very easy," with scores of 0 or 1 assigned, resulting in a total score between 0 and 16. Scores classify health literacy as inadequate (0-8), problematic (9-12), or sufficient (13-16). The Greek version showed excellent reliability (Cronbach’s α = 0.903) [33].

Procedure

This six-month cross-sectional study (March to July 2025) used convenience sampling. Most questionnaires (88%) were completed online via Google Forms, with the remaining 12% administered in person. The online survey employed a snowball sampling approach, initially inviting ten participants from the general Greek population. All participants agreed to take part and further disseminated the questionnaire, facilitating the recruitment of the target sample size.

Data analysis

Data were analyzed using the Statistical Product and Service Solutions (SPSS, version 29; IBM SPSS Statistics for Windows, Armonk, NY), and descriptive statistics were reported. Given the inconsistent replication of the HeLD’s seven-factor structure in previous studies [16,21,28], Exploratory factor analysis (EFA) was conducted. The dataset’s suitability for EFA was checked via the KMO measure and Bartlett’s test. Factor extraction was guided by eigenvalues >1, scree plot, parallel analysis, and a first-to-second eigenvalue ratio >4 to indicate unidimensionality [34]. Factor loadings ≥0.40 were considered significant. Internal consistency was evaluated using Cronbach’s alpha and McDonald’s omega, along with item-total correlations and alpha if an item was removed, with α ≥ 0.70 considered acceptable [35]. Concurrent validity was assessed by correlating HeLD-14 scores with the HLS-EU-Q16 using Pearson’s correlation. To address missing data, the “Exclude cases analysis by analysis” option was applied. A significance level of p < 0.05 was applied for all analyses.

Ethical considerations

Participants were fully informed about the study and gave voluntary, anonymous, and confidential consent. They could withdraw at any time without consequences, and online users consented by clicking a prompt. No compensation was offered. The study was approved by the Ethics Committee of the University of Thessaly’s Department of Nursing (approval number 65/2025).

## Results

Factor structure of the scale

EFA (principal axis factoring (PAF) with direct Oblimin rotation) was conducted to evaluate the underlying factor structure and assess the factorial validity of the scale. PAF was deemed appropriate for this study, as the primary objective was to identify the underlying latent constructs rather than merely to reduce data. Furthermore, Oblimin rotation was applied to allow for potential correlations among factors, a common and realistic assumption in social science research. This combination is widely utilized to explore the structure of complex constructs, such as oral health literacy, examined in the present study.

The Kaiser-Meyer-Olkin (KMO) measure (0.885) and Bartlett’s test of sphericity (χ² = 2052.205, p < 0.001) confirmed data suitability. Four factors were extracted (eigenvalues >1), explaining 54.7% of variance. Some items showed cross-loadings or low loadings, but the first-to-second eigenvalue ratio (5.836) suggested the scale is essentially unidimensional [34].

**Figure 1:**
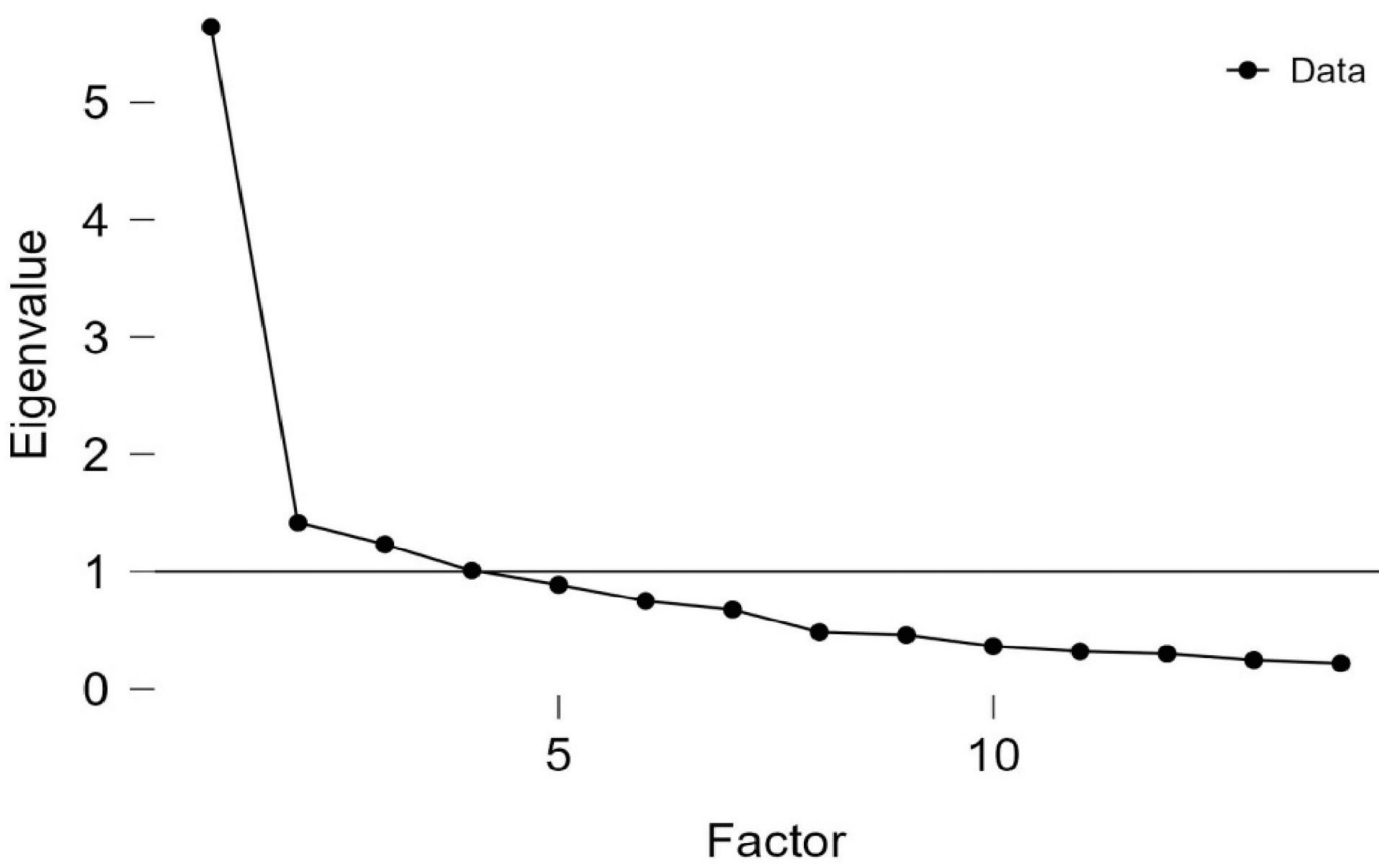
Screen plot

Consequently, based on this ratio and the scree plot (Figure 1), a one-factor solution was examined, accounting for 38% of the total variance. All items loaded ≥0.40, indicating strong associations with the latent construct of oral health literacy (refer to Table 2). Parallel analysis with 100 replications further supported the unidimensional structure of the scale. Item 12 had the highest loading, while Item 6 had the lowest. The one-factor solution was the most parsimonious and interpretable, confirming that the Greek HeLD-14 has a unidimensional construct.

**Table 2:** Factor loadings HeLD-14: Health Literacy in Dentistry Scale-14. Applied rotation method is oblimin.

HeLD-14 Items	Question	Factor 1	Uniqueness
Item 1	“Are you able to pay attention to dental health needs?”	0.680	0.537
Item 2	“Are you able to make time for things good for dental health?”	0.587	0.656
Item 3	“Are you able to fill in dental forms?”	0.551	0.697
Item 4	“Are you able to read dental information brochures?”	0.512	0.737
Item 5	“Are you able to take support to a dental appointment?”	0.619	0.617
Item 6	“Are you able to ask for support to a dental appointment?”	0.483	0.767
Item 7	“Are you able to pay to see a dentist?”	0.617	0.620
Item 8	“Are you able to pay for dental medication?”	0.649	0.578
Item 9	“Do you know how to get a dentists appointment?”	0.554	0.693
Item 10	“Do you know what to do to get a dental appointment?”	0.577	0.667
Item 11	“Are you able to look for a second opinion?”	0.562	0.684
Item 12	“Are you able to use information?”	0.747	0.441
Item 13	“Are you able to carry out dental instructions?”	0.704	0.504
Item 14	“Are you able to use dentists’ advice?”	0.716	0.487

Reliability analysis

The Greek HeLD-14 demonstrated good internal consistency, with Cronbach’s alpha = 0.879 and McDonald’s ω = 0.877. Item analysis showed that removing any item would not improve reliability (see Table 3).

**Table 3:** Reliability statistics

Estimate		McDonald’s ω			Cronbach's α
Point estimate		0.877			0.879
95% CI lower bound		0.857			0.859
95% CI upper bound		0.897			0.897
Reliability Statistics for individual items					
If item dropped					
Item	McDonald's ω		Cronbach's α	Item-rest correlation	
						HELD 1	0.861		0.866	0.643	
						HELD 2	0.867		0.870	0.562	
HELD 3	0.871		0.872	0.547	
HELD 4	0.873		0.874	0.504	
HELD 5	0.864		0.867	0.612	
HELD 6	0.876		0.878	0.463	
HELD 7	0.866		0.869	0.573	
HELD 8	0.864		0.867	0.608	
HELD 9	0.871		0.874	0.500	
HELD 10	0.871		0.874	0.519	
HELD 11	0.871		0.873	0.515	
HELD 12	0.871		0.869	0.665	
HELD 13	0.870		0.869	0.632	
HELD 14	0.869		0.868	0.640	

Participants scored relatively high on the Greek HeLD-14 (mean = 48.23, SD = 7.75, range: 5-56). The highest literacy was for Item 10 (“Do you know what to do to get a dental appointment?”), and the lowest for Item 2 (“Are you able to make time for things good for dental health?”). Skewness values suggested approximate normality for most items, though kurtosis values indicated some deviations (see Table 4).

**Table 4:** Descriptive statistics for each item of the HeLD-14 HeLD-14: Health Literacy in Dentistry Scale-14

Item	Valid	Missing	Mean	Std. Deviation	Minimum	Maximum	Range	kurtosis	skewness
HELD 1	333	0	3.186	0.913	0	4	4	0.693	-1.023
HELD 2	333	0	3.168	0.886	0	4	4	0.429	-0.937
HELD 3	333	0	3.210	1.116	0	4	4	1.545	-1.496
HELD 4	332	1	3.244	1.084	0	4	4	1.724	-1.541
HELD 5	333	0	3.459	0.900	0	4	4	3.385	-1.888
HELD 6	333	0	3.339	1.180	0	4	4	2.126	-1.811
HELD 7	333	0	3.363	1.876	0	4	4	1.200	-1.289
HELD 8	333	0	3.195	0.964	0	4	4	0.608	-1.111
HELD 9	333	0	3.850	0.582	0	4	4	23.417	-4.674
HELD 10	333	0	3.865	0.546	0	4	4	27.553	-4.995
HELD 11	332	1	3.361	1.070	0	4	4	2.641	-1.834
HELD 12	333	0	3.727	0.576	0	4	4	7.602	-2.473
HELD 13	333	0	3.700	0.596	1	4	3	4.290	-2.099
HELD 14	333	0	3.667	0.694	0	4	4	5.847	-2.379

Concurrent validity

Concurrent validity of the Greek HeLD-14 was supported by a significant positive correlation with the HLS-EU-Q16 (r = 0.424, p = 0.0008).

## Discussion

This study is the first to evaluate the Greek HeLD-14, examining its factor structure, internal consistency, and concurrent validity. The findings provide an initial foundation for further validation and comprehensive psychometric assessment in Greek-speaking populations.

The HeLD-14 was found to be unidimensional, with good internal consistency and concurrent validity. All items loaded ≥0.40, the highest for Item 12 (“Are you able to use information?”) and the lowest for Item 6 (“Are you able to ask for support for a dental appointment?”). This parsimonious, interpretable factor solution was retained for further analyses.

The Greek HeLD-14’s unidimensional structure aligns with findings from Jones et al. (HeLD-29 in Indigenous Australians) [4] and studies in the US and China [20,21]. However, it contrasts with prior HeLD-14 adaptations in Peru, Brazil, and Thailand, which showed multifactorial structures [19,23,26], and differs from the shortened HeLD-14, where six of seven original components were retained [16].

Differences in HeLD findings may result from varying analytical methods and diverse populations, including distinct cultural and socioeconomic contexts [4,16,21,23,28]. Such variability in dimensional structures is common when developing instruments to assess latent constructs [20].

The Greek HeLD-14 showed strong internal consistency (Cronbach’s α = 0.879; McDonald’s ω = 0.877), comparable to the original and Brazilian validations [16,19,21]. Although slightly lower than some Arabic, Peruvian, Chinese, and Colombian studies [21-25], it exceeds the reliability reported in other Arabic and Persian versions [36,37]. No items improved α if removed, confirming the scale’s consistency.

The Greek HeLD-14 demonstrated a significant positive correlation with the HLS-EU-Q16, consistent with findings reported in the Arabic and Chinese HeLD-14 validation studies [21,24]. Although the correlation was moderate, this may reflect differences between general and oral health constructs. Nonetheless, we consider this level of association sufficient to support adequate concurrent validity.

The Greek HeLD-14 had a mean score of 48.23 and a median of 50, similar to Arabic and Peruvian studies [23,24]. The Peruvian mean was slightly lower (47.47). Similar to the findings of the Arabic validation study, a potential ceiling effect was observed, likely attributable to the sample consisting predominantly of younger, middle-aged, and highly educated adults.

Unexpectedly, participants did not view economic barriers as a major obstacle to dental care, unlike findings from the Brazilian elderly cohort. The greatest difficulties were in the receptivity dimension, especially Items 1 and 2, a pattern also observed in other settings such as Peru [23].

Unlike Peruvian and Brazilian studies [19,23], normality assumptions were not fully met for all HeLD-14 items in the Greek sample.

The validated Greek HeLD-14 provides a tool for research and clinical practice, helping identify factors influencing oral health literacy and guiding policies and interventions, especially for vulnerable populations.

Additional research involving more diverse populations is needed to further investigate the dimensionality and psychometric properties of the HeLD-14. Future studies should employ methods such as confirmatory factor analysis and assess test-retest reliability, multiple forms of validity, and the potential influence of sociodemographic factors. The current findings provide preliminary evidence and underscore the need for continued investigation.

The study’s strength lies in being the first to evaluate oral health literacy in the general Greek population using the HeLD-14. Limitations of the study include the use of convenience sampling, which may limit generalizability; the absence of test-retest reliability assessment; and unknown response rates resulting from online distribution and self-selection. Furthermore, younger and highly educated individuals were overrepresented in the sample, and the online administration mode may have inflated health literacy scores.

## Conclusions

The HeLD-14 instrument demonstrates both validity and reliability for assessing multiple dimensions of oral health literacy among Greek-speaking adult populations. Its brevity and ease of administration, coupled with robust psychometric properties, make it a suitable tool for evaluating oral health literacy in oral public health research contexts. However, these findings are preliminary and should be confirmed in future studies.
